# P04.25. Complementary medicine use and potential adverse reactions amongst HIV-positive people

**DOI:** 10.1186/1472-6882-12-S1-P295

**Published:** 2012-06-12

**Authors:** L Braun, M Dooley, C Forrester, A Duncan, K Mackie

**Affiliations:** 1 Monash, Melbourne, Australia; 2 The Alfred, Melbourne, Australia

## Purpose

Relatively little is known about the patterns of complementary medicine (CM) use by HIV-positive people in the last decade, since the introduction of contemporary antiretroviral therapy (ART), or their information requirements. As ART use has evolved, CM use is likely to have also changed over time, however this has yet to be established. This is of relevance to health care providers, partly because of potential risks including drug interactions, but also to understand reasons for use and intended benefits, such as managing antiretroviral therapy (ART) side effects and/or addressing existing disease symptoms. The primary aim of this study is to establish the current patterns of use of CMs by HIV-positive people in Australia. Secondary aims are to identify their main information sources and the prevalence of potential drug interactions and suspected adverse reactions to CMs.

## Methods

A ten site multi-center study is being conducted at hospital and sexual health centre sites around Australia. Hospital pharmacists are recruiting patients at HIV outpatient clinics and dispensaries. The study aims to collect survey responses from a broad cross-section of over 1000 HIV-positive volunteers.

## Results

Data collection began in October 2012 and is due for completion by May 2012. Results from the Victorian sites will be available by February 2012.

## Conclusion

It is anticipated that this study will be the impetus for developing specific complementary medicine resources and education to assist healthcare providers in counseling HIV-positive people about the safe and appropriate use of CMs and in helping patients to select the appropriate treatment or therapy to utilize.

